# Continuous Glucose Monitoring as a Valuable Tool in the Early Detection of Diabetes Related to Cystic Fibrosis

**DOI:** 10.3389/fped.2021.659728

**Published:** 2021-07-09

**Authors:** Bojana Gojsina, Predrag Minic, Sladjana Todorovic, Ivan Soldatovic, Aleksandar Sovtic

**Affiliations:** ^1^Department of Pulmonology, The Institute for Health Protection of Mother and Child Serbia, Belgrade, Serbia; ^2^Faculty of Medicine, University of Belgrade, Belgrade, Serbia; ^3^Department of Endocrinology, The Institute for Health Protection of Mother and Child Serbia, Belgrade, Serbia; ^4^Institute for Medical Statistics and Informatics, Faculty of Medicine, University of Belgrade, Belgrade, Serbia

**Keywords:** cystic fibrosis related diabetes, continuous glucose monitoring, lung function decline, oral glucose tolerance test, hemoglobin A1c

## Abstract

**Aims:** We evaluated the impact of cystic fibrosis-related diabetes (CFRD) on lung disease and nutritional status.

**Study Design:** The retrospective cohort study evaluated the subjects' medical records from 2004 to 2019. All participants older than 10 years diagnosed by a 30-minutely sampled OGTT formed OGTT-CFRD subgroup. The participants diagnosed with continuous glucose monitoring (CGM) (at least two peaks above 11.1 mmol/l and more than 10% of recorded time above 7.8 mmol/l) formed a CFRD-CGM subgroup. The participants without CFRD formed a non-CFRD group. The longitudinal follow-up was made 2 years before and 3 years after insulin therapy initiation.

**Results:** Of 144 participants included, aged 10–55 years (44% males), 28 (19.4%) had CFRD. The HbA1c was significantly lower in the CGM-CFRD in comparison to the OGTT-CFRD subgroup (5.9 ± 0.62 and 7.3 ± 1.7% respectfully; *p* = 0.04). Subjects with CFRD were malnourished in comparison to non-CFRD, with significant improvements with insulin replacement therapy in regard to BMI Z-score (−1.4 ± 1.3 vs. −0.5 ± 1.2%, *p* = 0.04) and pulmonary exacerbation score (*p* = 0.02). In OGTT-CFRD subgroup there is an increase in FEV1 (62.7 ± 26.3 to 65.1 ± 21.7%, *p* = 0.7) and decrease in FVC (from 76.4 ± 24.2 to 71.2 ± 20%, *p* = 0.003) from diagnosis to second year of follow-up. In CGM-CFRD subgroup there was a decrease in FEV1 (from 58.2 ± 28.2 to 52.8 ± 25.9%, *p* = 0.2) and FVC-values (from 72.4 ± 26.5 to 67.4 ± 29.1%, *p* = 0.08).Chronic *Pseudomonas aeruginosa* infection was more prevalent in the CFRD group (*p* = 0.003).

**Conclusion:** Continuous glucose monitoring is a useful tool for insight of glucose impairment and diagnosis of CFRD. Early recognition of CFRD and therapeutic intervention has favorable effects on clinical course of the disease.

## Introduction

Cystic fibrosis (CF) is the most common lethal autosomal recessive disorder in non-Hispanic White with a prevalence of 1: 2,500–5,000 live births (1). Many factors, especially early diagnosis, hypercaloric diet, and aggressive treatment of exacerbations, have led to a shift in patients' median life expectancy (2). Prolongation of life led to increased prevalence of cystic fibrosis-related diabetes (CFRD) (1, 3). Disorders in glucose metabolism can also occur in younger age (in 2% of children), but the majority of patients are diagnosed with CFRD during adolescence or in adults (in up to 40% of all cases) (4). The pathophysiological mechanism of CFRD is complex. It is primarily caused by insulin deficiency, but unlike diabetes mellitus type 1, β-cell damage in CF is not of autoimmune origin. Abnormal chloride channel function results in thick viscous secretions that lead to obstructive damage to the exocrine pancreas. Destruction of islet architecture is due to chronic inflammation, progressive fibrosis and fatty infiltration (5). Besides insulin insufficiency, insulin resistance also plays a role (6). There is peripheral insulin resistance, which arises from decreased glucose uptake by muscle, and hepatic insulin resistance, which is caused by impaired suppression of hepatic glucose production (7). Insulin resistance can get worse with acute pulmonary exacerbation, severe chronic lung disease and systemic glucocorticoid therapy (5).

What has been described as asymptomatic insulopenia in early childhood, if unrecognized, over the years lead to progressive malnutrition, loss of lung function, and increased number of exacerbations with a significant negative impact on survival and quality of life. Although its importance is validated and presented in official recommendations, the true prevalence of CFRD in CF centers varies, probably due to disagreement on age when screening is performed and different methodologies and diagnostic criteria in use^1^. In the last decade, the novel method for the early detection of CFRD has been suggested—continuous glucose monitoring (CGM) (8). Although it is not currently approved as diagnostic tool for CFRD by official position statements, its complementary use to OGTT leads to better prediction of symptomatic insulin deficiency and urges earlier treatment initiation. It was showed that the early stages of insulin deficiency may be contributing to catabolism and malnutrition, promoting lung bacterial growth and deteriorating lung function in CF. As the primary mechanism of CFRD is insulin deficiency, insulin replacement therapy is needed (9).

The aim of our research was to evaluate the impact of CFRD on lung disease, nutritional status and frequency of exacerbation. We hypothesized that early recognition of CFRD with CGM and prompt initiation of insulin therapy, was related to the less gradual decline of lung function, fewer pulmonary exacerbations and improvements in patients' nutritional status.

## Methods

In this retrospective cohort study, data extracted from patients' history files from January 2004 until December 2019, were evaluated. All participants were treated in the national CF center—Mother and Child Health Institute of Serbia. Data included demographics, underlying diseases, nutritional status, CFRD evaluation, sputum microbiology, frequency of exacerbation, and lung function testing. Subjects were in clinically stable condition, at least 1 month apart from pulmonary exacerbation, without concomitant use of systemic glucocorticoid therapy. The 5 years follow-up period, covered time span of 2 years before and 3 years after the diagnosis. For the purpose of this research, an exacerbation was defined as a worsening of respiratory symptoms that required oral or intravenous antibiotic therapy.

### CFRD Evaluation

The CFRD group consisted of subjects with diagnosis confirmed either by 30-minutely sampled oral glucose tolerance test (OGTT) or CGM (when become available), in asymptomatic patients on regular annual check-ups from 10 years of age (9). Participants with fasting hyperglycemia (above 7.0 mmol/l) were scheduled for further evaluation, no matter when screened previously.

The OGTT screening was performed using 1.75 g/kg of body weight of glucose up to maximum of 75 g. The blood glucose and insulin levels were measured over a period of 2 h at 30-min intervals. The participants were considered to have CFRD if the glycemia at 120 min was ≥11.1 mmol/l (OGTT-CFRD subgroup) (10).

A subgroup of subjects in whom CGM was used in diagnostic algorithm besides OGTT was presented separately. The CGM (available in our center from 2015), was performed within 3 months after OGTT in each subject, using the device applied outpatiently (Medtronic iPro^TM^2 Professional CGM system). The device stayed *in situ* for 7 days in the home environment for all subjects. They were on their usual diet and being advised to measure a minimum four self-monitored blood glucose levels daily. They were advised keeping a diary for glucose measurements and dietary intake. The participants were considered to have CFRD based on interstitial blood glucose levels on CGM, if there were at least two peaks above 11.1 mmol/l and more than 10% of recorded time above 7.8 mmol/l (CGM-CFRD subgroup). The non-CFRD group consisted of all other CF patients older than 10 years, without CFRD. Glycated hemoglobin (HbA1c)-values were measured for every subject on each occasion, when OGTT was performed. Value of HbA1c at diagnosis in CFRD group and last obtained value in non-CFRD group, were considered to be important for the analysis.

Nutritional status for each year of follow-up was expressed as a Z-score of body-mass index (BMI). Pancreatic sufficiency was proved with the absence of symptoms of maldigestion, combined with normal values of fecal elastase. For the purpose of this study, CF liver disease was defined as palpation of an enlarged liver and/or spleen or changes different than fatty infiltration on routine annual assessment ultrasound.

Chronic use of inhaled corticosteroids (ICS) in low/moderate doses (<400 mcg/day of budesonide or equivalent) in year preceding the CFRD assessment, was registered for each patient. None of the patients were treated with chronic systemic corticosteroid therapy 3 months preceding baseline.

### Lung Function

All study participants performed forced spirometry, according to official ERS guidelines (11) using a volume-constant method (MasterLab, Jaeger, Würzburg, Germany). The reference equations used for pulmonary function testing were those of Zapletal et al. (12). In the CFRD group the central event was the year when the diagnosis of CFRD was established. The best annual values of forced expiratory volume in the first second (FEV_1_) and forced vital capacity (FVC) in each year of follow-up were taken to calculate the 5-year trend value of these parameters. For the non-CFRD group, the best annual values of FEV_1_ and FVC in the last 5 years of follow-up were considered for the analysis.

### Statistics

Statistical data were evaluated by IBM SPSS Statistics 25 software program and were expressed as median and range. The choice of statistical test depended on the data type and distribution—parametric (ANOVA of repeated measurements), non-parametric (Chi-square test, Fisher's exact test). Correlations were evaluated by Pearson's and Spearman's rank correlation tests. To describe the relationship of two or more variables, linear regression was used.

The observation of lung function values trend, frequency of exacerbations and nutritional status was performed as well. In subjects with CFRD, central event was the insulin replacement therapy initiation. The trends of abovementioned parameters were calculated having in mind 2-year period preceding the CFRD diagnosis and 3-years of follow-up. In subjects from the non-CFRD group, last 5 years of follow-up was considered to be of interest, when calculating such a trend.

A statistically significant difference was considered to be *p* < 0.05. The study protocol was approved by the local Ethics committee (decision number 8/2). The research was conducted according to Declaration of Helsinki.

## Results

The study included 144 subjects, 28 (19.4%) subjects in the CFRD group and 116 (80.6%) in the non-CFRD group. The mean age at diagnosis of CFRD was 20.7 ± 9.6 years. The mean age in the OGTT-CFRD subgroup was 21.0 ± 8.8 years, and in CGM-CFRD subgroup 25.9 ± 8.7 years. In all patients in whom CGM measurement confirmed the diagnosis of CFRD, OGTT results were inconclusive for the diagnosis of CFRD. The average value of HbA1c in the CFRD group was 6.8 ± 1.6%, which was comparable to values from the non-CFRD group-−6.4 ± 1.2%. In subjects from the OGTT-CFRD subgroup, the value of HbA1c was significantly higher than in the CGM-CFRD subgroup (7.3 ± 1.7 and 5.9 ± 0.62% respectfully, *p* = 0.04). A careful reassessment of the patient's medical data did not show an association of possible comorbidities or therapeutic interventions and HbA1c levels. For the participants in the CGM-CFRD group, maximal measured glucose levels were from 13.5 to 15.2 mmol/l. In 12–33% of recorded time on CGM, glycemia was above 7.8 mmol/l, with three to seven peaks above 11.1 mmol/l.

The comparable number of subjects from CFRD and non-CFRD groups was homozygote for F508del mutation. Four pancreatic sufficient subjects from non-CFRD group, proved to be compound heterozygote with residual function mutation. Demographic characteristics are shown in Table 1.

**Table 1:** Demographic data.

	**CFRD**	**Non-CFRD**	**Total**	***p***	**CGM-CFRD**	**OGTT-CFRD**	**Total**	***p***
Number	28	116	144		7	21	28	
Male, *n* (%)	13 (46.4)	51 (43.9)	64 (44.4)	0.84	4 (57.1)	9 (42.9)	13 (46.4)	0.67
Female, *n* (%)	15 (53.6)	65 (56.1)	80 (55.6)	0.84	3 (42.9)	12 (57.1)	15 (53.6)	0.67
Age	20.7 ± 9.6	18.6 ± 9.2	19.7 ± 9.4	0.62	25.9 ± 8.7	21.0 ± 8.8	23.5 ± 8.7	0.32
Age at CF diagnosis	3.2 ± 3	2.7 ± 3.63	2.8 ± 3.3	0.91	3.6 ± 2.9	2.8 ± 3.1	3.2 ± 3	0.74
Age at CFRD diagnosis	20.7 ± 9.6	/	20.7 ± 9.6	/	25.9 ± 8.7	21.0 ± 8.8	23.5 ± 8.7	0.32
Pancreatic insufficiency, *n* (%)	28 (100)	112 (96.6)	140 (97.2)	1	7 (100)	21 (100)	28 (100)	/
Liver disease, *n* (%)	19 (67.8)	44 (37.9)	63 (43.7)	**0.01**	5 (71.4)	14 (66.7)	19 (67.8)	1
FEV_1_ 2 years prior to baseline, (%)	66.1 ± 22.9	84.7 ±23.7	75.4 ± 23.3	**0.001**	57.5 ± 23.6	69.7 ± 23	66.1 ± 22.9	0.06
FEV_1_ at baseline, (%)	61.6 ± 26	82.4 ± 24.4	72.8 ± 25.3	**0.001**	58.2 ± 28.2	62.7 ± 26.3	61.6 ± 26	0.81
FEV_1_ at second year of follow up, (%)	57.7 ± 25.5	77.8 ± 27.1	67.8 ± 26.3	**0.001**	52.8 ± 25.9	65.1 ± 21.7	57.7 ± 25.5	0.06
FVC 2 years prior to baseline, (%)	77.4 ± 19.6	86.7 ± 17.8	82.1 ± 18.7	0.06	69.2 ± 22.5	81.2 ± 18	77.4 ± 19.6	0.05
FVC at baseline, (%)	76.3 ± 23.9	85.3 ± 17.9	80.8 ± 20.9	0.06	72.4 ± 26.5	76.4 ± 24.2	76.3 ± 23.9	1
FVC at second year of follow up, (%)	70.9 ± 21.7	83.1 ± 19.6	77 ± 20.6	**0.04**	67.4 ± 29.1	71.2 ± 20	70.9 ± 21.7	0.9
Homozygote for F508del mutation, *n* (%)	14 (50)	64 (55.2)	78 (54.1)	0.82	4 (57.1)	10 (47.6)	14 (50)	0.84
BMI Z-score 2 years prior to baseline (mean)	−1.2 ± 1.1	−0.4 ± 1.2	0.8 ± 1.1	0.05	−1.3 ± 1.1	−0.9 ± 1.3	−1.2 ± 1.1	0.8
BMI Z-score at baseline (mean)	−1.4 ± 1.3	−0.5 ± 1.2	0.9 ± 1.3	**0.04**	−1.7 ± 1.4	−1.2 ± 1.4	−1.4 ± 1.4	0.4
BMI Z-score at second year of follow-up (mean)	−1.1 ± 1.4	−0.6 ± 1.2	0.8 ± 1.3	0.07	−1.4 ± 1.1	−1 ± 1.5	1.1 ± 1.4	0.4
PA infection, *n* (%)	21 (75)	51 (44)	72 (50)	**0.003**	6 (85.7)	15 (71.4)	21 (75)	0.64
BC infection, *n* (%)	4 (14.3)	14 (12.1)	18 (12.5)	0.75	0 (0)	4 (19)	/	0.54
SA, *n* (%)	4 (14.3)	24 (20.7)	28 (19.4)	0.58	0(0)	4 (19)	/	0.54
Chronic ICS use	16 (57.1)	34 (29.3)	50 (34.7)	**0.01**	6 (85.7)	10 (47.6)	16 (57.1)	**0.02**
HbA1C (%)	6.8 ± 1.4	6.4 ± 1.2	6.6 ± 1.4	1	5.9 ± 0.62	7.6 ± 1.7	6.8 ± 1.4	**0.04**
Glycemia in 60 min during OGTT (mmol/l)	11.1 ± 2.3	8.5 ± 2.0	9.8 ± 2.5	0.27	9.1 ± 2.3	12.9 ± 2.3	11.1 ± 2.3	0.31
Glycemia in 120 min during OGTT (mmol/l)	9.1 ± 0.9	7.8 ± 2.5	8.3 ± 3.2	0.82	5.6 ± 0.6	12.5 ± 1.3	9.1 ± 0.9	**0.02**

*CFRD, patients diagnosed with CFRD; CGM-CFRD, subgroup diagnosed with continuous glucose monitoring; OGTT-CFRD, subgroup diagnosed with oral glucose tolerance test; CF, cystic fibrosis; DM, diabetes mellitus; PA, Pseudomonas aeruginosa; BC, Burkholderia cepacia; SA, Staphylococcus aureus, FEV_1_, forced expiratory volume in 1 s. Bold values represent statistically significant P < 0.05*

### Nutritional Status and Liver Disease

**Figure 1:**
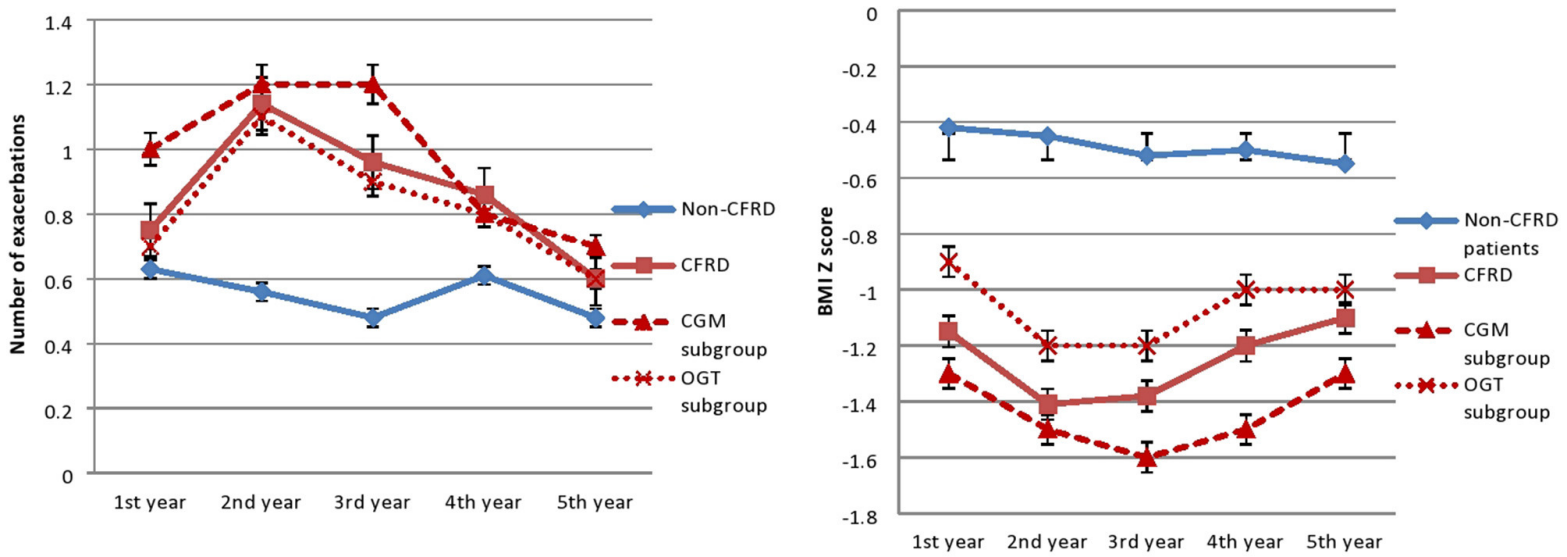
Number of exacerbations and BMI Z-score. In the CFRID group the number of exacerbations significantly decreased (*p* = 0.02), and BMI Z-score significantly improved (*p* = 0.04) after initiating insulin therapy in the third year of follow-up.

In the year when diagnosis was confirmed, subjects from the CFRD group were significantly more malnourished in compare to the non-CFRD group (−1.4 ± 1.3 vs. −0.5 ± 1.2, *p* = 0.04). In addition, weight gain on insulin replacement therapy resulted in significant improvement in BMI Z-score in CGM-CFRD subgroup (from −1.7 ± 1.4 to −1.4 ± 1.1, *p* = 0.02), but not in the OGTT-CFRD subgroup (from −1.2 ± 1.4 to −1 ± 1.5, *p* = 0.07)—Figure 1, Table 1.

Data analysis showed that liver disease was present more frequently in CFRD group (*p* = 0.007). It had been shown that participants with liver disease had lower FEV_1_-values (*p* = 0.03).

### Lung Function and Pulmonary Exacerbations

The inhaled ICS were used more frequently in the CFRD group (*p* = 0.001). In the CFRD group, the number of exacerbations decreased significantly after initiating insulin therapy (*p* = 0.02). The number of exacerbations didn't change significantly in the non-CFRD group (*p* = 0.5)—Figure 1.

**Figure 2:**
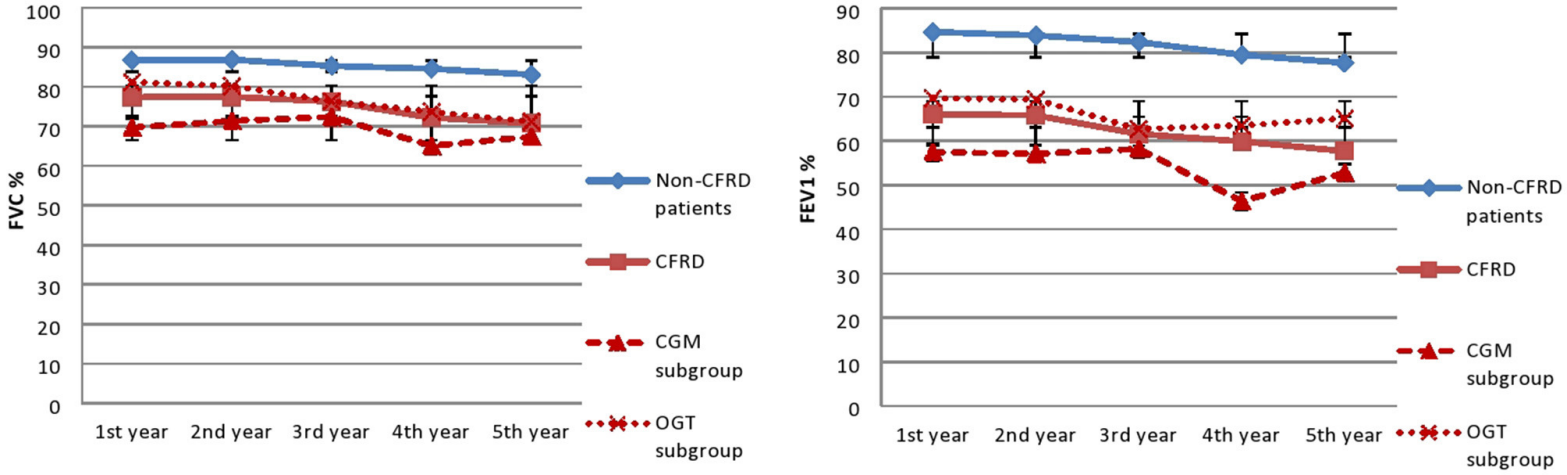
Lung function trends. FVC, forced vital capacity; FEv_1_, Forced expiratory volume in 1 s. In third year insulin therapy was started. There is a difference within each group during the observed period (*p* < 0.001), no significant difference was observed between groups.

Participants from CFRD group have significantly lower FEV_1_ compared to non-CFRD group (61.6 ± 26 and 82.4 ± 24.4% respectfully, *p* < 0.001). Using correlation analysis it was showed that FEV_1_ positively correlates with BMI (*r* = 0.610, *p* < 0.001) and negatively correlates with age (*r* = −0.559, *p* < 0.001) The 5-year trend values of lung function was examined and there was a statistically significant negative trend of FEV_1_- and FVC-values over time in both groups (*p* < 0.001)—Figure 2.

As for the CFRD group, in OGTT-CFRD subgroup there is an insignificant increase in FEV_1_ (from 62.7 ± 26.3 to 65.1 ± 21.7%, *p* = 0.7) and decrease in FVC (from 76.4 ± 24.2 to 71.2 ± 20%, *p* = 0.003) from diagnosis to second year of follow-up. In CGM-CFRD subgroup there was a decrease in FEV_1_-values (from 58.2 ± 28.2 to 52.8 ± 25.9%, *p* = 0.2) and FVC-values (from 72.4 ± 26.5 to 67.4 ± 29.1%, *p* = 0.08) at the year of diagnosis and in the second year of follow-up—Figure 2. But there is no difference between CGM-CFRD and OGTT-CFRD subgroups in the second year of follow up in regard to FEV_1_ and FVC (52.8 ± 25.9 vs. 65.1 ± 21.7%, *p* = 0.06 and 67.4 ± 29.1 vs. 71.2 ± 20%, *p* = 0.9, respectively)—Table 1.

Chronic colonization of the lower respiratory tract with *Pseudomonas aeruginosa* was more frequent in the CFRD group compared to the non-CFRD group (*p* = 0.003). However, the incidence of chronic colonization with *Burkholderia cepacia* and *Staphylococcus aureus* was not significantly different between groups.

## Discussion

We showed that the occurrence of CFRD has an important, unfavorable effect on the course of CF. This is reflected in more severe malnutrition, a further lung function impairment and frequent exacerbations of CF lung disease. In addition, we showed that CGM monitoring is useful in diagnosis of CFRD, even though OGTT results were not confirmative.

Regular blood glucose control is an essential part of evaluating response to therapy and adjusting treatment regimens, but is not suitable because in requires frequent measurements. Although the study was not designed to evaluate sensitivity of CGM in the diagnosis of CFRD, the earliest glucose abnormalities are seen with it. This was also confirmed by the results of other research, where more than half of CF patients with normal glucose tolerance by OGTT demonstrate intermittent postprandial glucose levels above 11.1 mmol/l (6, 13). Hameed et al. found that having glucose level above 7.8 mmol/L for ≥4.5% of the time during CGM predict a greater decline in BMI Z-score and FEV_1_ (5). Respiratory dysfunction correlates with the degree of insulopenia and the severity of glucose metabolism disorders (7). Interestingly, a beneficial effect was present in recent studies with the improvement of lung function parameters during insulin therapy (1). We confirmed previously published data showing that presence of CFRD negatively correlates with lung function and nutritional status (4, 14, 15). Chan et al. showed a negative correlation between CGM results (peak glucose, excursion above 7.8 mmol/l and percentage of time above 7.8 mmol/l), lung function, and BMI Z-score (16). Moreover, the results of our research showed that early diagnosis of CFRD no matter which diagnostic method was used, followed by the initiation of insulin therapy, had beneficial effects on lung function decline, frequency of exacerbations and BMI Z-score, regardless of patients' age. Although it is believed that HbA1c levels ≥6.5% is consistent with diabetes (5, 6), its levels were not higher in the CFRD group in compare to non-CFRD. The levels of HbA1c were lower in the CGM-CFRD subgroup, meaning that the normal HbA1c-value does not exclude CFRD. In addition, it was showed that some subjects from non-CFRD group had HbA1c levels ≥6.5%, but normal CGM and OGTT data. HbA1c is generally thought to underestimate hyperglycemia in CF, but not necessarily to overestimate average glycemia in this population. These results confirmed the limited significance of HbA1c in the diagnosis of CFRD, but do not preclude its significance in disease follow-up.

Since CFTR chloride channel defect plays a role in the β-cell and in insulin secretion, CFTR modulator therapy currently in use and new drugs in the pipeline, might impact CFRD prevalence in years to follow. In recent study of Gaines et al. (17) 36% of CFRD patients treated with CFTR modulators had markedly improved disease status. Several patients developed persistent hypoglycemia after CFTR modulator therapy initiation, probably due to relatively delayed reduction of insulin therapy. The beneficial effects of such therapy are probably related to its anti-inflammatory potential. Some other chronic anti-inflammatory therapy (e.g., oral azithromycin) has beneficial effects on the CF lung disease, but such effects of ICS have not been established, except for patients with associated asthma (18). Although the results of our research showed that ICS administration was significantly more common in patients with CFRD, it had been shown before that this therapy at low to median daily doses had no effect on glucose metabolism (5). So, it seems reasonable to believe that ICS therapy in CF should be used cautiously.

Our research has shown that CFRD is more common in patients with liver disease, which may be related to the complex mechanisms of glycoregulation that take place in the liver. Although pancreatic insufficiency (PI) is associated with a more severe clinical features, its representation in both groups is similar, which is different from the large studies that suggest positive correlations between CFRD and PI (19). However, the prevalence of PI should not be neglected because it reached 100% in the CFRD group, suggesting that the number of subjects was insufficient to achieve a significant statistical difference. Disease severity is unpredictable when mutations from two different classes are present, but mild CF phenotype is mostly present in subjects with at least one residual function mutation (mutation classes IV–VI). Four pancreatic sufficient patients from non-CFRD group and none from CFRD group proved to have at least one residual function mutation. In addition, susceptibility for development of CFRD in PI patients is in part influenced by genetic variants in alternative chloride channels and single-nucleotide polymorphisms for type 2 diabetes (20) which were not analyzed for the purpose of this research.

Dysregulation of glucose metabolism promote bacterial growth in the lungs and result in more frequent pulmonary exacerbations, with further impairment of respiratory function and life expectancy, as confirmed by the results of our study (5). We showed that chronic *P. aeruginosa* infection is more prevalent in the CFRD group. These results were comparable to the research of Leclerq et al. (13), although their definition of CFRD was less strict than we used in our study. In CF patients, airway mucous glucose levels become elevated at systemic glucose levels >8 mmol/l and these glucose-containing secretions are associated with growth of respiratory pathogens (6). Undiagnosed CFRD in patients chronically colonized with *P. aeruginosa*, may accelerate lung function decline despite adequate chronic suppressive antimicrobial therapy.

This study has several limitations. The sample is relatively small, which may interfere with the results and evaluation of the examined associations. Additional work is needed to demonstrate the CGM-thresholds at which insulin intervention may be beneficial. In addition, the study is limited to a single hospital location. Its strengths are in a timely evaluation of CFRD using CGM, underling its usefulness as a possible more accurate and earlier indicator of dysglycemia than the 2-h blood glucose on OGTT. It also included longitudinal follow-up, after the initiation of insulin therapy.

We believe early diagnosis of CFRD should be encouraged in regular clinical practice. It has beneficial effects due to timely initiation of insulin therapy that result in better glycoregulation, improved nutritional status and lung function, fewer pulmonary exacerbations and prolonged life expectancy. Regular CGM should be used complementary with OGTT and HbA1 in order to have better insight in patient's CFRD status. Finally, the authors would suggest that CGM criteria should be included in CFRD Clinical Care Guidelines in nearest future.

## Data Availability Statement

The original contributions presented in the study are included in the article/supplementary materials, further inquiries can be directed to the corresponding author/s.

## Ethics Statement

The studies involving human participants were reviewed and approved by Ethics Committee Mother and Child Health Institute. Written informed consent to participate in this study was provided by the participants' legal guardian/next of kin.

## Author Contributions

BG and AS contributed for literature search, data collection and organization of database, study design, statistical analysis, manuscript preparation, and manuscript revision. IS contributed for data collection and organization of database, statistical analysis, and manuscript revision. ST contributed for literature search, manuscript preparation, and manuscript revision. PM contributed for manuscript preparation and manuscript revision. All authors contributed to the article and approved the submitted version.

## Conflict of Interest

The authors declare that the research was conducted in the absence of any commercial or financial relationships that could be construed as a potential conflict of interest.

## Footnotes

^1^ECFS Patient Registry (https://www.ecfs.eu/ecfspr).
